# Eight Letters with Replies

**Published:** 1879-07

**Authors:** 


### Our Correspondence.

THORNTON, TEXAS, April 16, 1879.
DEAR DOCTOR:—

I have in my possession a stone which is said to be the *mad stone.* It was taken from the stomach of a white deer. I have applied it in two cases where children were bitten by a rabid dog. Its behavior was very singular. To one of the children it attached nine times, to the other six times, after which it would take hold no longer. The time of filling was fifteen to twenty minutes. The dog that bit these children bit two other dogs and some hogs, all of which died of hydrophobia in twenty days. It has now been a year, and the children are still alive. Have applied it to the bite of one snake. The stone attached three times with perfect relief to the little sufferer. I have lately applied it to a gentleman's hand who was working a horse that had hydrophobia and got some of the saliva on his hand. The stone would fill every half hour for twenty-four hours and then refused to stick any longer. These facts can be established by as creditable authority as any in the State. The stone is porous, light, and of a brown color. Can you account for its singular behavior, or why these parties have not died from hydrophobia?

I have often heard of the mad stone, but this is the first and only one I have ever seen.

Respectfully,
JAMES R. MILLS, M. D.

The probable solution of the action of the "mad stone," is that it is an *anhydride* compound, light and porous. To cause such a substance to adhere, the skin must be moistened. Water coming in contact with an anhydride substance changes it into an acid, that *may* neutralize the poison. The rapid absorption of moisture from the skin, filling the pores of the stone, causes it to adhere by atmospheric pressure. You now have a cupping machine which *may* extract the virus or perhaps bring it in contact with the acid that *may* neutralize it.

---

MENDON, ST. JO. CO., MICH., Jan. 6, 1878.
DR. UP DE GRAFF:

It is impossible to estimate in money the value of such rich "contributions," such gems of research and chrystals of experience, as one gathers up in your little quarterly, the BISTOURY. It can only be afforded on the principle stated by Mr. Towner in the last number, "selfsatisfaction of doing good work if you do not get a cent for it." The leading article, *Honor Bright*, is just the thing for the times, and should be copied into secular papers.

In response to the bill sent for a half dollar I send a dollar. Yours truly,

EDWIN STEWART, M. D.

That is what might be styled practical encouragement. Thanks for your good words, Doctor; you are booked for the next two years.

---

BALTIMORE, MD., April 12, 1879.
DR. UP DEGRAFF:

*Dear Sir:*—The BISTORY came to hand in due time "and contents noted," particularly that boy Fritz. My youngster (Percy) wants to know if he allowed the poor little gold fish to die, when he could so easily have put them back into the water? This seems to trouble him considerably. A. J. H. W.

Calm little Percy's fears by assuring him that the gold fish were soon restored to their native element, where they disport themselves as usual. They seem to keep a sharp eye on Fritz, however, whenever he ventures into the conservatory.

---

A LETTER CHOCK FULL OF PUNS.

The editor of this journal recently had a birth-day frolic, to which a few of his most intimate friends were invited, by means of a sort of pictorial card. A well known contributor to the BISTOURY, residing in a neighboring city, received one of these cards, to which he replied in the following manner, to wit:

April 10, 1879.
MY D. R. DOCTOR:

Your pictorial stationery has been picked o'er, and as I decipher its meaning, I do sigh for a chance to go for tea to your fortieth birthday par T; for T., S. U. and yours spread it, is ever nice, but nicer still would it be to score myself one at your two score affair. I k. k. k., that is I kan't kome kause I can't get away. So you are forty! What an older codger you are getting to be! But I was forty, four years ago, and am only forty-four yet. So I am stationary, but not as pictorially so as your's. I'm sorry I can't pen an inkling of how I feel about this affair of yours, having no pen and ink to do it with; but in Pennsylvania we don't consider a pencil vain, ye see, as I am lead to remark. [If this point is dull, whittle it.]

May you live forty years more! and may every year be happier than its predecessor! As you draw near the apex of your life, the acclivity up which you have come can be looked down upon without many regrets. I trust, my dear fellow, that your pathway down the thither side of life may prove even more free from perplexities than that up the hither side. A portion of this, we have travelled in company with each other. Whether any portion of that will find us again comrades, no one may know. But of this be ever assured, that though leagues separate us materially, in thought, in memory, in imaginings, I am often near, even with you. I wish you joy of your birthiday fete, and joy of the friends you will gather around you on the occasion. Think of me as rejoicing for you if not with you, and believe me as ever,

Yours exceedingly, BEN.

---

CENTREVILLE, AMITE CO., MISS., April 14, 1879.

THAD S. UP DEGRAFF, M. D.:

*Dear Sir:*—I have long since given up the practice of medicine, but last summer at Chattanooga, I got sight of some numbers of your journal and was so pleased with it that I sent for the volume of 1878, and subscribed for the current year. Although I no longer practice, I yet feel considerable interest in medicine, and as I am living on my place here in the country, I have not kept posted on the new medicines. As your journal has frequent reference to medicines I never used and know nothing of, I write to ask you what book I would better get that will give me the needed information. I do not care to go to much expense, such as getting a new edition of Wood & Bache, yet as a matter of satisfaction and to enable me better to appreciate your admirable little journal, I want you to give me the name and price of such a work as will suit me.

Very respectfully yours,
J. R., M. D.

We do not know of any cheap work on materia medica, such as the doctor calls for. Perhaps some of our professional readers do, and will acquaint us with it.

The best work we know anything about is, Therapeutics and Materia Medica, by Alfred Steele, M. D., Henry C. Lea, Phila., publisher. There are two volumes costing $10.

---

STANDISH, MICH., April 2, 1879.

THAD S. UP DEGRAFF, M. D.:

*Dear Sir:*—Please give me your experience as to the best mode of administering salicylic acid in acute rheumatism. I have used it only once and that recently in a case I had here, with good results. I gave the dry powder in wafers, fifteen grains every hour until the patient was relieved. The dose, however, is too bulky to swallow to advantage.

I notice in the July number of the BISTOURY for 1876, an article on "the rapid cure of acute rheumatism with salicylic acid." The writer states that the pure acid is soluble in water. It will not dissolve the acid I have.

Yours respectfully,
J. M. OLDFIELD, M. D.

The article, salicylate of soda in acute rheumatism, in present number of the BISTOURY will answer the doctor's query. Dissolve half ounce of bi-carbonate of soda in eight ounces of water. To this add your salicylic acid in such quantity as is desired. It will dissolve.

---

MT. ANDREW, ALA., April 17, 1879.

DR. THAD S. UP DEGRAFF:

*Dear Sir:*—Enclosed please find fifty cents subscription to the BISTOURY for the present year. Although I have never been a subscriber I have been an occasional reader of the BISTOURY, and regard it as one of the very best journals I have ever read. It is always a welcome visitor at every fireside where it is taken in this country. It contains, not only many valuable hints and prescriptions to the fireside reader, but to the physician as well. I notice a remedy for catarrh of the bladder and ammoniacal odor of the urine, by Dr. E L. Bertherand, the Arenaria rubra, that I would like very much to get as I have a patient under treatment with that disease. At times the odor is so intolerable that he avoids society all together. Please do me the favor to inform me where the Arenaria Rubra can be procured, how to prepare it, the dose, etc., and you will confer a special favor, not only on myself, but my patient. Very truly, W. R. M.

We wrote Wm. R. Warner & Co., Phila., who inform us that they were unable to obtain the *arenaria rubra* in this country. Perhaps some of our readers may apprise us of where it can be had.

---

INDIANAPOLIS, IND., April 30, 1879.

DR. THAD S. UP DE GRAFF:

*Dear Doctor:*—I send you a sample of "Mr. J. A. Drollinger's opium cure." It is made and sold in Laporte in this State. I strongly suspect it contains morphia. Can you tell?

J. D. F., M. D.

The sample sent proves to be glycerine colored with aniline red and containing ¼ per cent. of sulphate of morphia. A beautiful "substitute," that, for opium.

